# Comparison of Computational Methods for Simulating Depolymerization Reaction

**DOI:** 10.1021/acsomega.4c09953

**Published:** 2025-02-04

**Authors:** Shunsuke Mieda

**Affiliations:** Platform Laboratory for Science & Technology, Asahi Kasei Corporation, 2-1 Samejima, Fuji, Shizuoka 416-8501, Japan

## Abstract

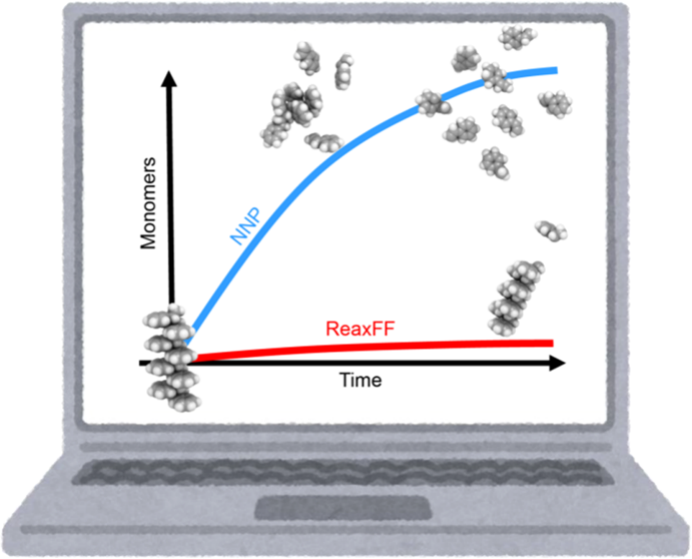

A chemical recycling process that reduces polymers to their raw materials plays a crucial role in circular economy. To contribute to chemical recycling, this study proposes a system that simulates the process of depolymerization from polymer-to-monomer using reactive molecular dynamics (MD). Two MD methods, Reax force field (ReaxFF) and neural network potential (NNP), were employed to simulate the depolymerization of a polystyrene model. We validated the simulation accuracies by comparing monomer yields and decomposition products with experimental results. The results showed that NNP-MD accurately replicated the degradation and redecomposition processes and achieved consistency with the experimental data at various temperatures. ReaxFF-MD, however, was less able to represent the depolymerization process. We conclude that NNP-MD is capable of simulating polymer depolymerization results that are consistent with experimental observations. These results contribute to the development of methods for efficient chemical recycling and the broader realization of a circular economy.

## Introduction

The circular economy (CE), which is a concept used to promote sustainable development, is gaining attention in various fields.^1^ We believe that the realization of CE is crucial for industry and for continued development in the future. Closing the materials loop is one of the requirements for realizing CE.^2^ For this reason, efforts have been made to recycle materials.^3−7^ However, the recycling rate of plastic, which is a man-made material that is quite stable and does not decompose easily in nature, is only about 9%.^8^ The disposal of plastics has a great environmental impact; thus, improving its recycling rate has become a major issue.^9^ At present, mechanical recycling, which involves melting and reforming plastics, is widely employed as a plastic recycling technology. However, mechanical recycling causes contamination of the plastics by foreign matter and subsequent degradation as well as the cross-linking and cutting of their polymer chains by thermal shock. Furthermore, thermosetting plastics cannot be remelted and have limited application.^10,11^ Therefore, chemical recycling, in which polymers are converted back to monomers, must be developed to achieve a CE.^11−13^

Chemical recycling consists of several elements. Among them, the depolymerization process, which reduces polymers to raw materials, is extremely important. Depolymerization technology has been studied for a considerable time. A variety of techniques have been proposed, such as chemolysis, pyrolysis, and gasification,^12^ and some polymers are chemically recycled industrially.^13^ However, as pyrolysis and gasification offer little benefit in terms of CO_2_ emissions, continued research on controlled depolymerization reactions using catalysts and solvents are needed.^9^ Catalyst design often involves identifying a rate-limiting reaction and lowering its activation energy by elucidating its mechanism. However, it is also known that the depolymerization reaction mechanism of even a simple polystyrene, for example, is quite complex.^14,15^ Therefore, we expect that elucidating the depolymerization reaction mechanism of commonly used copolymers and resins that contain additives would be difficult.^16,17^

Therefore, we propose that a system capable of simulating the depolymerization reaction and automatically clarifying its polymer-to-monomer-formation reaction mechanism is needed. The systems that automatically simulate reactions are known, such as the global reaction route mapping (GRRM)^18^ and the reactive molecular dynamics (MD) method. GRRM is a powerful tool for exploring the reaction pathways between reactants and products, but it cannot easily simulate a large system that includes entire polymers. For example, to simulate polymerization, one study applied GRRM to some reactions in collaboration with nonreactive Monte Carlo and MD, which do not cause chemical reactions during simulation.^19^ Therefore, we adopt a method that uses reactive MD for the depolymerization of the entire polymer.

MD methods based on frequently used molecular force fields, such as polymer consistent force fields,^20^ typically cannot simulate reactions because such force fields require prior acquisition of bonding information and do not account for bond switching during simulations. Therefore, to conduct reactive MD, an energy evaluation method that either does not rely on bonding information or can flexibly modify bonding information during the simulation is required. Such reactive MD methods include Ab Initio MD (AIMD),^21^ Reax force field (ReaxFF)-MD,^22^ and neural network potential (NNP)-MD.^23^ MD simulation using Density Functional Theory (DFT-MD), which is a kind of AIMD, calculates the energies and forces at each step by using DFT calculations. DFT calculations are known to be highly accurate, but also highly computationally expensive and difficult to carry out on a large scale. NNP-MD computes energies and forces at each step by using NNP calculations, which potentially exhibit an accuracy level comparable to that of DFT while incurring <1/100 of the computational cost of DFT.^24^ Given that an NNP is a model trained on the results of quantum chemical calculations, such as DFT, to predict energies and forces based on atomic structures, an inadequately trained NNP may yield inaccurate results. To ensure reliable outcomes, updating the NNP through retraining during simulations^25,26^ or using pretrained NNP models adequately trained on a vast data set of atomic structures is essential. ReaxFF is a molecular force field in which bond order is determined by the distance between atoms and ReaxFF-MD represents bond formation and dissociation. However, the computational cost of DFT-MD is quite high, which limits its simulations to the order of 10 ps for systems containing a few hundred atoms, a number that is clearly insufficient for simulating polymer degradation. However, ReaxFF-MD and NNP-MD, which are reactive MD and not computationally expensive compared to DFT-MD, perform simulations on the order of 100 ps or more. Molecular degradation^27−30^ and depolymerization^31−35^ have also been reported. However, no simulated polymer-to-monomer depolymerization reactions have achieved quantitative agreement with the experimental results.

Therefore, the purpose of this study is to verify whether polymer-to-monomer decomposition can be simulated by conducting reactive MD simulations using ReaxFF or NNP. The simulations are validated by comparing monomer yields and the types and amounts of byproducts produced with experimental results. Furthermore, methods for performing efficient depolymerization reactions are discussed. This simulation focuses on thermal depolymerization, which can occur with the polymer alone. Polystyrene, a polymer that undergoes thermal decomposition and whose decomposition products have been extensively studied, including the quantity of byproducts, was used as the target polymer.

## Results and Discussion

### Polymer Degradation by a Polystyrene Model

**Figure 1:**
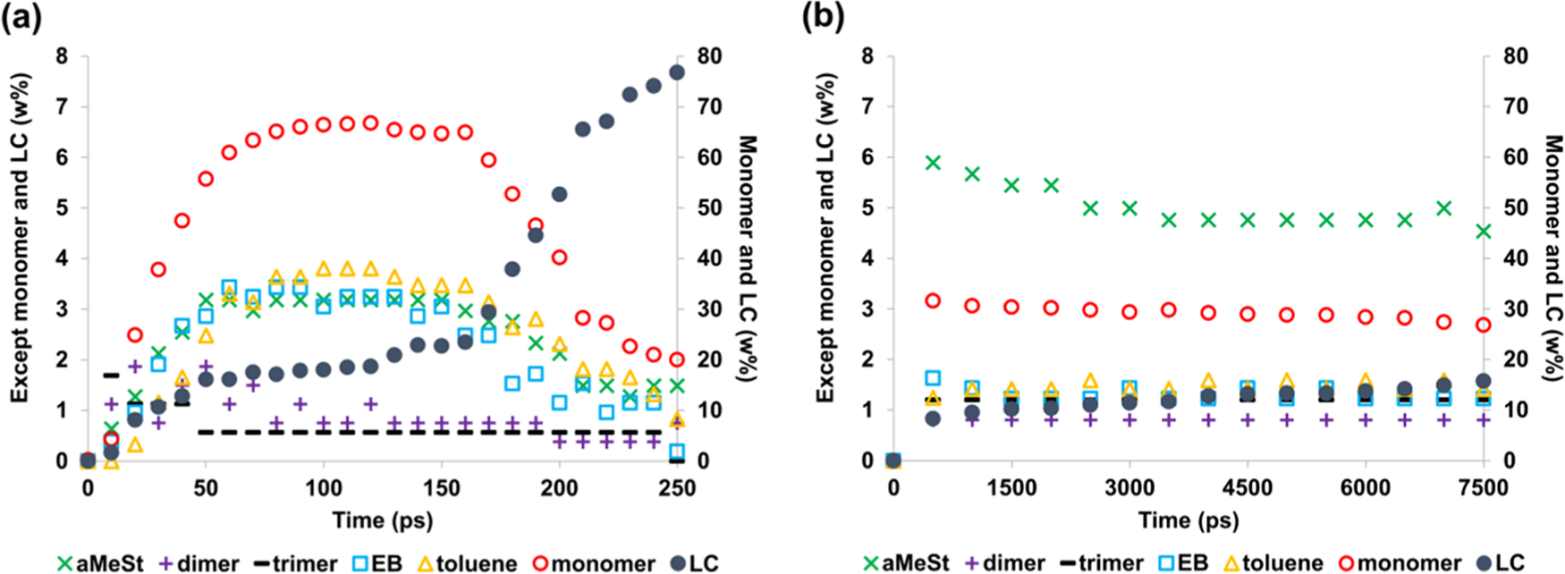
Decomposition products of polystyrene at 2000 K obtained by using (a) NNP-MD simulation and (b) ReaxFF-MD simulation. The horizontal axis represents simulation time, and the vertical axis represents the weight percentage of degradation products present.

The degradation behavior of the polystyrene at 2000 K is shown in Figure 1. The vertical axis of the figure shows the weight percentage of degradation products present in the system, where the percentages of monomer and light hydrocarbon (LC) are on the right, and the percentages of other byproducts are on the left. The horizontal axis shows the change over time in units of picoseconds. Figure 1(a,b) shows the results for NNP-MD and ReaxFF-MD, respectively. Figure 1(a) shows that the weight fraction of monomer in NNP-MD reaches as high as 66.7 wt % but begins to decrease around 160 ps, whereas the weight fraction of LC increased. This indicates that the monomer produced by decomposition is redecomposed at the high temperature of 2000 K.^36^ As styrene monomer has been shown to decompose at temperatures above 1500 K in experiments,^37^ our simulation results are consistent with previous results. Moreover, the weight percentages of other decomposition products, such as ethylbenzene (EB) and toluene, also tend to decrease over time, which suggests that these decomposition products were also redecomposed. However, the trimer appears to be nearly undecomposed; only one trimer was produced over the 20 trials, and it was assumed that this trimer was the one that remained undecomposed by chance. Figure 1(b) shows that for ReaxFF-MD, the monomer percentage reached up to 30 wt %, but there was no subsequent decrease in the weight percentage of monomer due to redecomposition. None of the other decomposition products showed significant changes after 100 ps.

The reason why the monomer fraction did not increase above 30 wt % during the ReaxFF-MD degradation simulation was thought to be the balance between redecomposition and monomer formation, but as LC did not increase either, this possibility is unlikely. Secondary reactions, as described in Guo et al.’s study of the pyrolysis of polypropylene by ReaxFF,^31^ could also be a reason, but as the structure at 7.5 ns was not significantly different from that of polystyrene (Figure S1), we conclude that this possibility is also unlikely. We do not think that these secondary reactions occur in ReaxFF-MD, but instead that the decomposition has simply stopped midway. The ranking of the weight percentage of the byproducts from both NNP-MD and ReaxFF-MD was monomer, LC, and other decomposition products. In NNP-MD, α-methylstyrene (αMeSt), EB, and toluene furnished almost the same ratio of decomposition products, whereas in ReaxFF-MD, αMeSt had the highest ratio, followed by EB and toluene, and then the ratio was lower. Direct comparison with experimental results was difficult because 2000 K is quite a high temperature. There is no experimental trend that is higher for only αMeSt, as far as I know.

**Figure 2:**
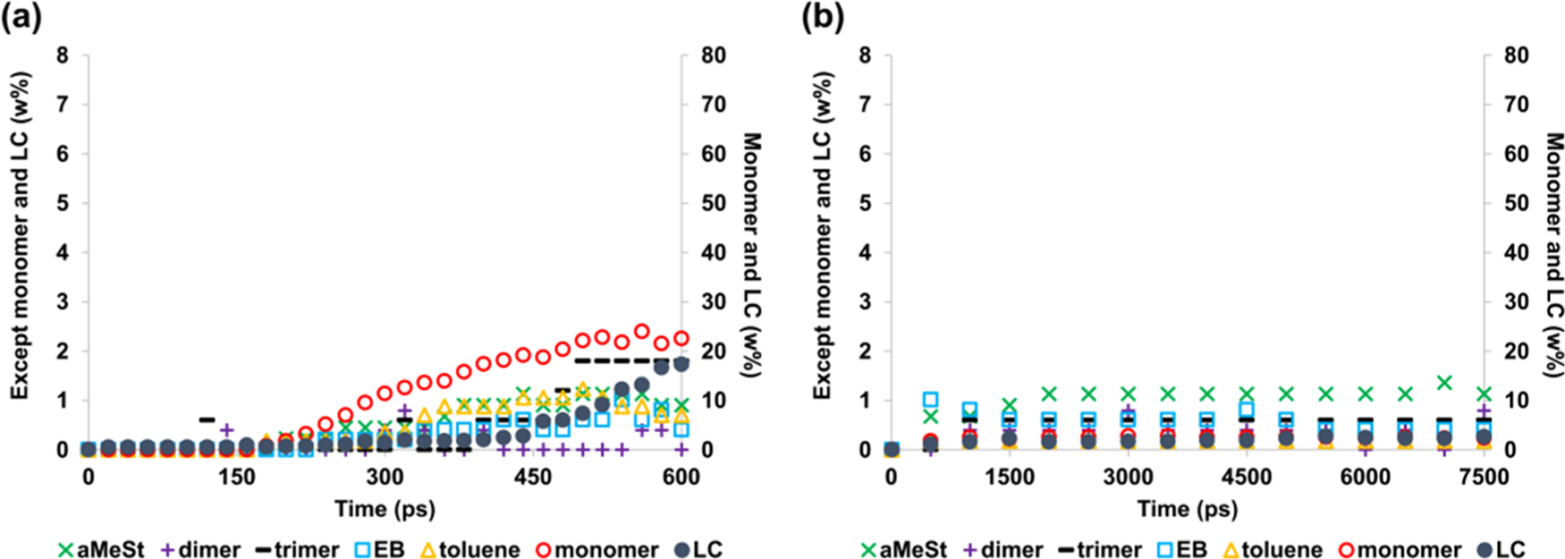
Decomposition products of polystyrene at 1500 K obtained by using (a) NNP-MD simulation and (b) ReaxFF-MD simulation.

The degradation behavior of the polymers at 1500 K is shown in Figure 2. Both NNP-MD and ReaxFF-MD show degradation rates that are clearly lower than at 2000 K (Figure 1). In particular, ReaxFF-MD showed almost no degradation, whereas NNP-MD began to degrade after 200 ps. However, at 600 ps, the monomer fraction was 21.8 wt %, which was not a sufficiently high weight percentage to indicate that degradation had adequately progressed. The structure of NNP-MD showed two distinct structures—one that degraded and one that did not (Figure S2). The sampling of the degraded system alone showed that the monomer fraction had reached almost 70 wt % (Figure S3). Even at 1500 K, the formation of radicals took a long time, so we thought it necessary to allow the radicals to form from the beginning to bring the simulation in line with the reality of lower temperatures.

### Polymer Degradation by a Radical Polystyrene Model

**Figure 3:**
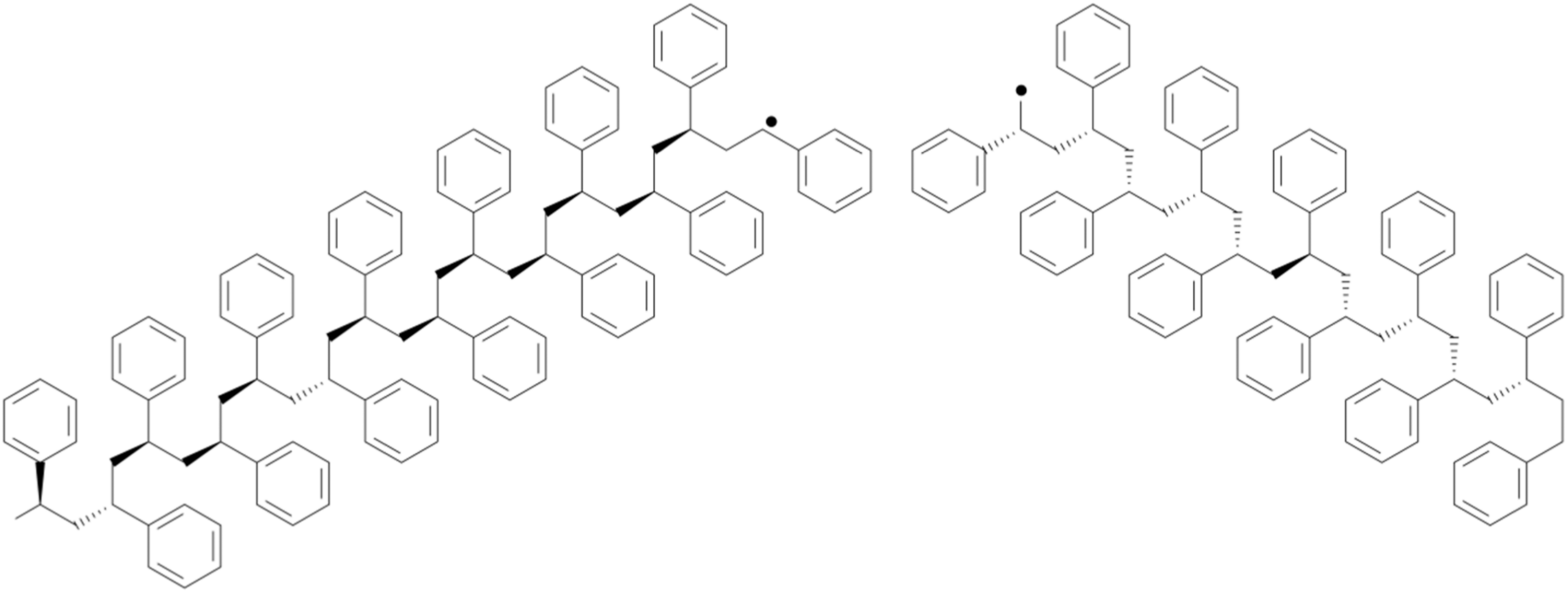
Radical structure as a starting point for thermal decomposition of polystyrene.

**Figure 4:**
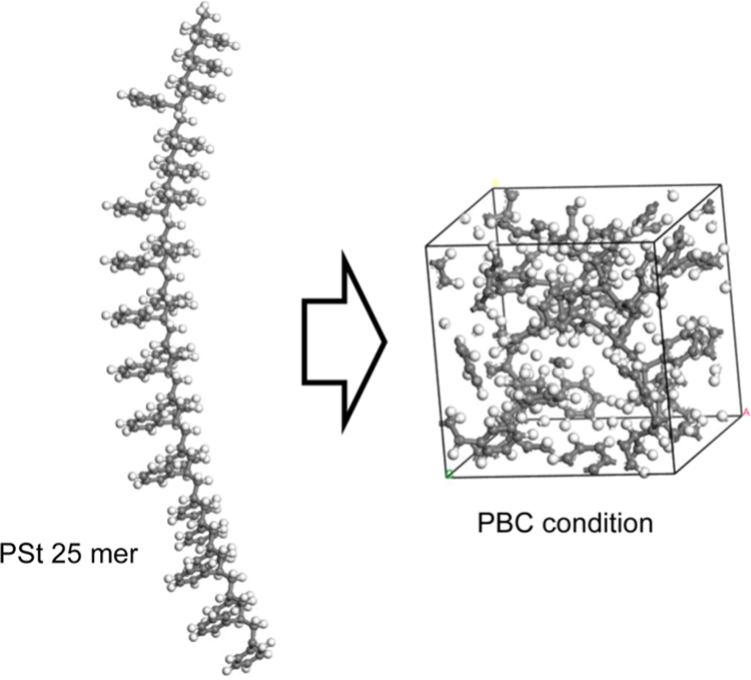
Atactic polystyrene 25-mer model with periodic boundary conditions used in this study.

In the simulations described above, the initiation of degradation was often observed from the dissociation of CC bonds in the polymer main chain, as shown in Figure 3. A similar degradation initiation mechanism has been proposed in experiments;^15^ thus, calculations were carried out using a structure that had single radical present at the terminal secondary carbon to simulate the reaction after CC bond dissociation. We refer to the model containing a radical in its initial structure as the radical polystyrene model. The model size was set to 25 mers, as shown in Figure 4.

**Figure 5:**
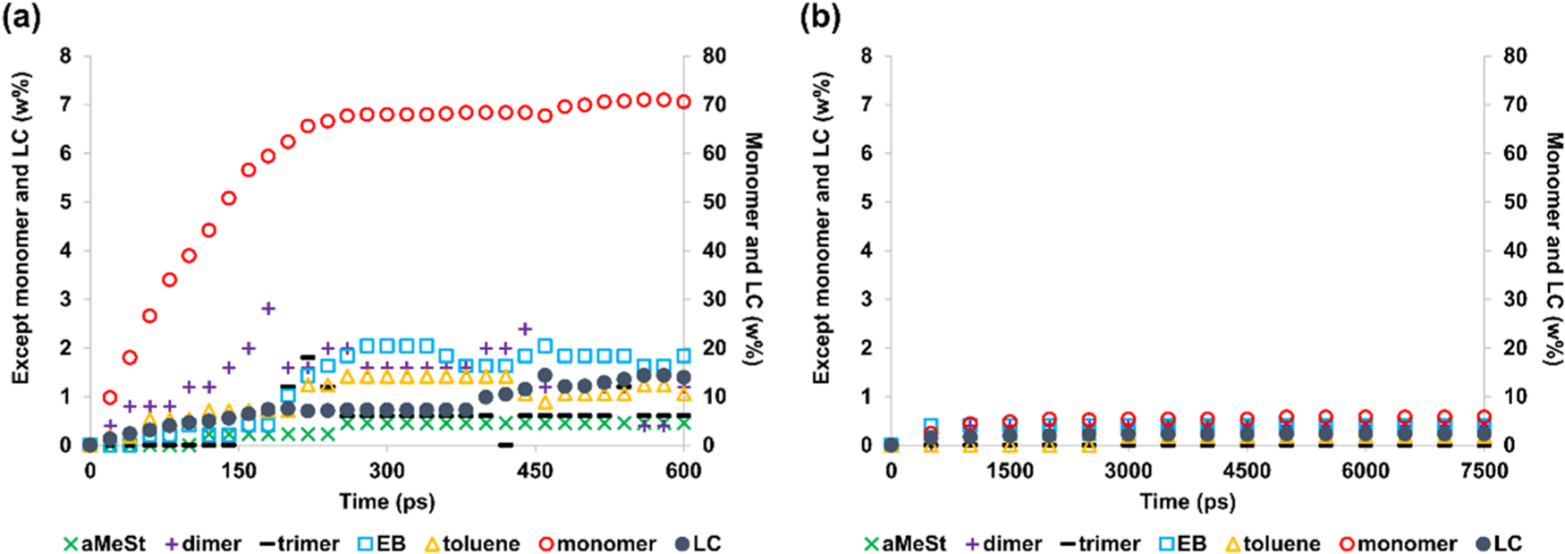
Decomposition products of polystyrene, which include a radical in the initial structure, at 1500 K obtained by (a) NNP-MD simulation and (b) ReaxFF-MD simulation.

Figure 5 shows the simulation results at 1500 K for the initial structure after the formation of radicals. For the NNP-MD shown in Figure 5(a), the monomer fraction reaches 70.6 wt % at 600 ps. As mentioned earlier, styrene monomer is known to redecompose at 1500 K. However, within the time scale of this simulation, no clearly observable extent of redecomposition occurred. Similarly, in the simulation using only the monomer, NNP-MD did not indicate monomer decomposition at 1500 K (Figure S4). In the presence of radicals or, to be more precise, with even one H atom reduced, ReaxFF-MD (Figure 5(b)) decomposed only as much as in the absence of radicals (Figure 2(b)).

**Figure 6:**
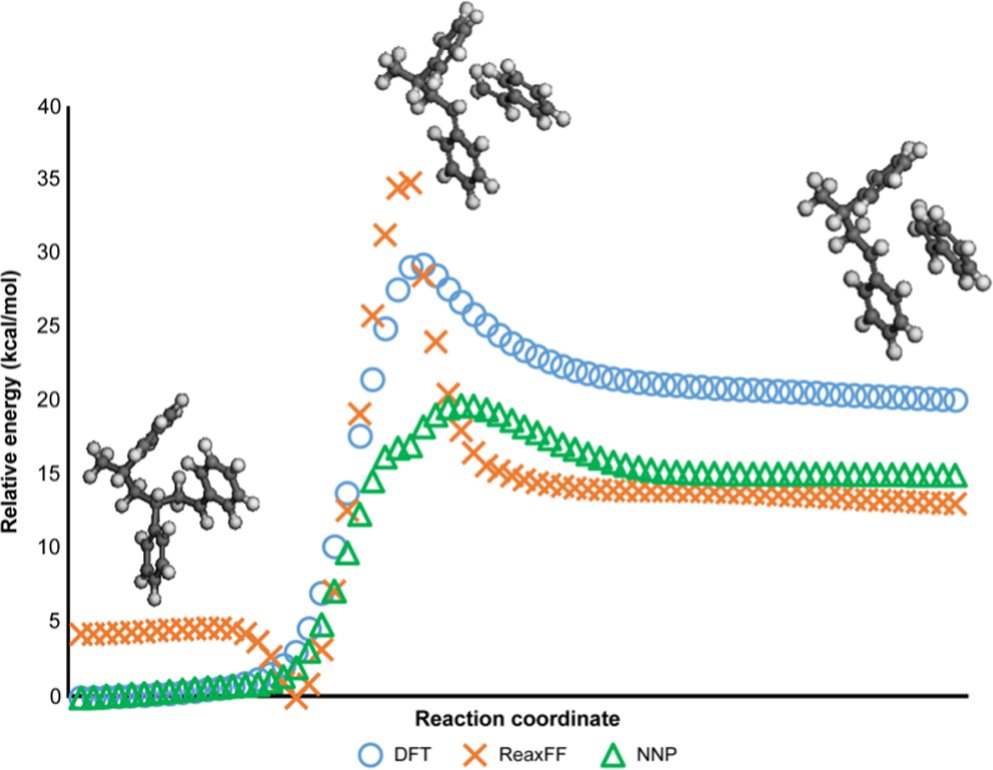
Coordinates for the reaction of terminal radical migration associated with desorption of the terminal monomer obtained by DFT, ReaxFF, and NNP.

Although the ReaxFF calculated in this study reproduced very well the behavior of bond dissociation, this was because it did not adequately represent the reaction of terminal radical migration that is associated with the desorption of the terminal monomer as in this case. However, the PreFerred Potential (PFP) has used the energies and forces calculated by DFT for various structures for training sets,^37^ so it can be used to calculate reactions such as this with good accuracy. As an example of this hypothesis, we calculated the monomer desorption reaction using a short polystyrene model with a radical present. We then compared the DFT results for the reaction to the results obtained using ReaxFF and NNP (Figure 6).

Figure 6 shows the reaction coordinate on the horizontal axis and the relative energy with respect to the most stable position on the vertical axis. According to Figure 6, NNP has lower activation energy than does DFT. However, the continuous increase in energy from the initial structure to the transition state (TS) is consistent with DFT. Additionally, the gradual decrease in energy until monomer desorbs after the TS formation is similar to DFT. We believe that this lower reaction energy and the similarity of the potential energy profile accelerated the MD simulation, enabling accurate decomposition within a short time scale. However, while accurate decomposition products were obtained for polystyrene, similarly precise results for other polymers cannot be guaranteed. In contrast, ReaxFF demonstrates different behavior, with the energy initially decreasing along the pathway from the initial structure to the TS, followed by a rapid increase and then a sharp drop after passing the TS. Furthermore, the energy of the TS is higher than that of DFT. This result also indicates that the ReaxFF used in this study did not represent the desorption reaction of the terminal monomer. Unlike a simple bond dissociation reaction generating two radicals, the monomer desorption reaction in the presence of terminal radicals requires the simultaneous formation of a π bond with the terminal radical and cleavage of the monomer σ bond. We believe that the ReaxFF potential employed herein does not adequately capture this orbital transformation. Nevertheless, achieving depolymerization in ReaxFF by employing strategies such as lowering the reaction energies, as in NNP-MD, and/or introducing a bias potential, as in hyperdynamics, may be possible.^38^

**Figure 7:**
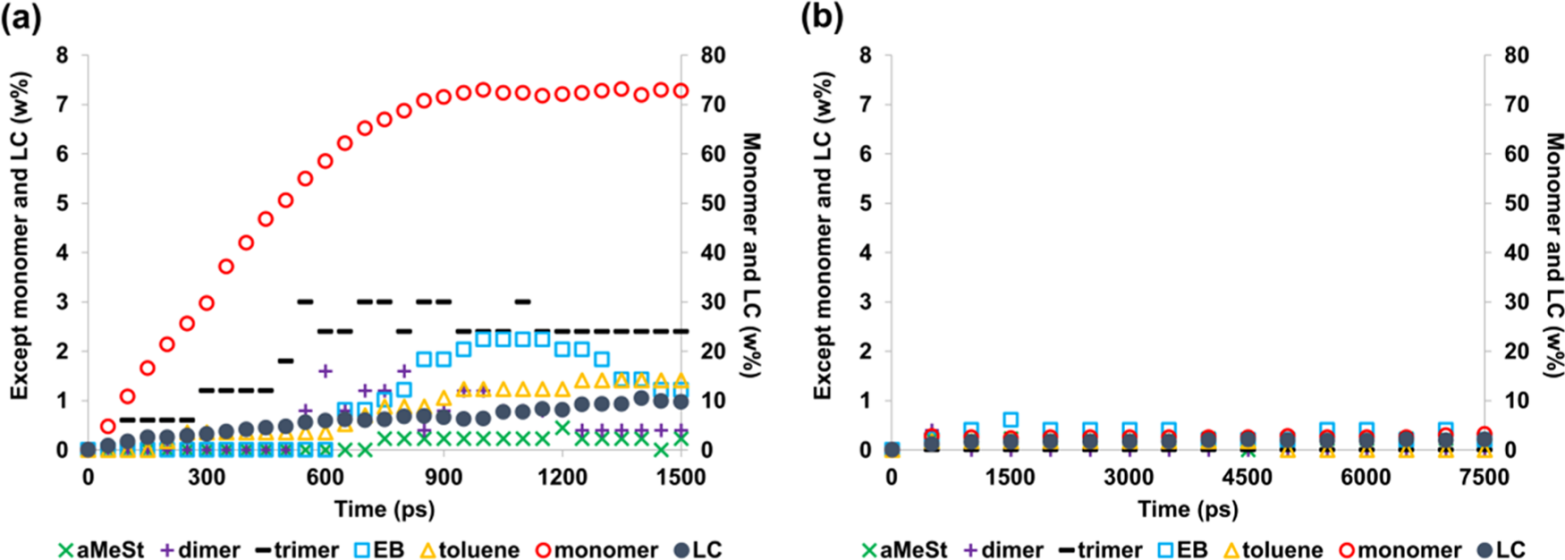
Decomposition products of polystyrene, which include a radical in the initial structure, at 1200 K obtained by (a) NNP-MD simulation and (b) ReaxFF-MD simulation.

We also performed degradation simulations at the low temperature of 1200 K (Figure 7). As shown in Figure 7(a), the monomer fraction of NNP-MD reached 72.7 wt % at 1500 ps. Table 1 shows the simulation and experimental results for the weight percentage of monomer LC, and the byproducts. Table 1 shows that the proportion of styrene at 1200 K is 72.7 wt % in the simulation, whereas it is 75.4 wt % at 1173 K and 70.0 wt % at 1248 K in the experiment by Bouster et al.^39^ Although the weight percentage of LC deviates somewhat from the experimental values, the definition of LC is not clear in the experiment, and thus, this result may stem from a definitional issue. The results for the decomposition products other than LC were found to be in good agreement with the experimental results. From these results, it can be concluded that NNP-MD can be used to simulate the temperature range within which the experiments are performed. However, the ReaxFF-MD results shown in Figure 7(b) and Table 1 did not decompose nearly as well as they did at 1200 K, even after 7.5 ns of simulation. Therefore, it was considered necessary to change the parameters or the formulation in order to simulate the decomposition behavior in ReaxFF-MD.

**Table 1:** Ratio of Decomposition Products Obtained by Depolymerization of Polystyrene in Weight Percentage^a^

	experiment^39^			NNP-MD	ReaxFF-MD
pyrolysis products	1073 K	1173 K	1248 K	1200 K	1200 K
styrene (monomer)	78.4	75.4	70.0	72.7	3.2
dimer	1.9	0.4	0.2	0.4	0.0
trimer	1.3	0.5	0.1	2.4	0.0
toluene	0.9	1.4	2.1	1.4	0.0
ethylbenzene	0.3	0.6	0.6	1.2	0.2
α-methylstyrene	0.9	0.9	1.1	0.2	0.2
light hydrocarbons	3.0	3.9	5.6	9.7	2.2

a Figure 7(a) presents the NNP-MD results at 1500 ps, whereas Figure 7(b) shows the ReaxFF-MD results at 7500 ps.

### Correlation between Degradants

**Figure 8:**
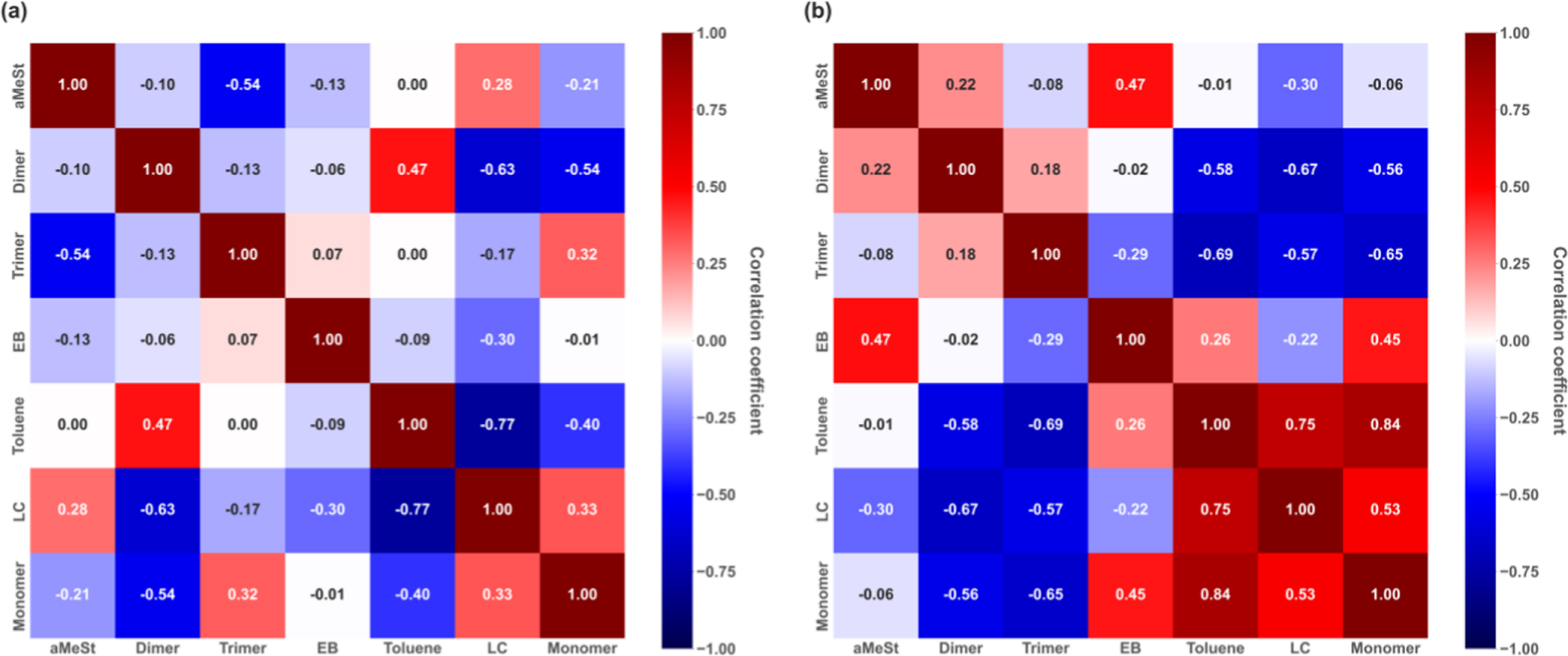
Correlations between the depolymerization products of polystyrene whose initial structure included a radical, obtained by NNP-MD simulation (a) at 1500 K and (b) at 1200 K. The white numbers represent correlation coefficients. Blue tiles represent negative correlations, and red tiles represent positive correlations.

Figure 8 shows the correlations between the degradants obtained from the NNP-MD simulation, where the vertical and horizontal axes represent the decomposition products and the correlation coefficients are marked on the intersecting tiles. Each tile is color-coded according to the value of its correlation coefficient. Figure 8(a) shows the correlation coefficients between the degradation products at 1500 K after 240 ps, in which the weight percentage of monomer obtained by decomposition no longer changes significantly. Figure 8(b) shows the correlation coefficients between the decomposition products at 1200 K after 900 ps.

Although listing all elementary reactions can be useful for understanding reactions occurring within a system, when dealing with highly complex reactions, such as depolymerization, clarifying correlations between individual products may be easier than listing all reactions. Hence, Figure 8 illustrates the correlations between the depolymerization products. In Figure 8(a), the absolute values of the correlation coefficients are low for most of the decomposition products at 1500 K; however, some correlations are evident. Negative correlations were observed between toluene and LC, dimer and LC, dimer and monomer, and trimer and αMeSt. These findings suggest potential direct or indirect reaction pathways between these molecules. For instance, although a negative correlation exists between the monomer and the dimer, recombination of the monomer with the dimer has not been observed. Instead, the dimer potentially decomposes into the monomer at 1500 K. According to the correlation coefficient of decomposition at 1200 K that is shown in Figure 8(b), many combinations show correlation. For example, as with 1500 K, dimer showed a negative correlation with monomer and LC. However, the trimer, demonstrating almost no correlation at 1500 K, exhibits a negative correlation with the monomer and LC at 1200 K. This is not because the trimer did not decompose at 1500 K but due to its yield, which was extremely low at 1500 K, was adequate at 1200 K, confirming its correlation owing to its decomposition (Figure S5). This means that trimers and dimers can become monomers by redecomposition, while pyrolysis can produce LC as a byproduct. In addition, positive correlations among EB, toluene, LC, and the monomer suggest that these products may be generated simultaneously.

## Conclusions

To successfully simulate polymer depolymerization and to realize chemical recycling, we simulated the degradation behavior of polystyrene using NNP-MD and ReaxFF-MD. We found that both methods showed a certain degree of degradation under the high temperature condition of 2000 K, and in particular, NNP-MD showed some redecomposition. At 1500 K, radical generation with which to initiate degradation did not occur easily for either method, and degradation did not progress satisfactorily. For this reason, simulations were carried out by generating radicals at the ends of the main chains under the assumption that the main chains were bonded and dissociated. The results showed that little degradation occurred for ReaxFF-MD, as occurred in the case of no radicals. However, for NNP-MD, decomposition occurred quickly even at 1500 K. Decomposition also proceeded at 1200 K for NNP-MD and was in very good agreement with the experimental data. The ReaxFF simulation used in this study has previously been used for a variety of degradation reactions, including polymers, but it is difficult to describe in cases where the position of the terminal radical shifts with the observed monomer desorption, such as in monomer desorption reactions.

The simulations with NNP-MD showed that the byproducts obtained in the temperature range below 2000 K were in good agreement with experiments. However, as calculations on the order of nanoseconds were necessary even at 1200 K, various acceleration methods had to be applied for studies at even lower temperatures or for long simulation times. The parameters and formulations for ReaxFF-MD must be improved to represent decomposition in monomer desorption reactions. Alternatively, it would be best to apply it in a system where bond dissociation mainly occurs at high temperatures. By utilizing simulation technologies to develop catalysts, we aim to apply them to polymers that are difficult to depolymerize, such as thermoplastics, and to achieve depolymerization at lower energies to achieve the chemical recycling of a wide range of polymers.

## Methods

Reactions with parts other than neighboring residue, such as intra- and intermolecular radical rearrangements, are important in the depolymerization process. Thus, a model of a certain size is needed. In this study, an atactic polystyrene 25-mer single chain was used as the model (shown in Figure 4), and an amorphous structure was created under periodic boundary conditions (PBC) using Amorphous Cell Tools in the Materials Studio software. Relaxation calculations were performed according to Hofmann’s method^40^ using the Forcite module in Materials Studio. The simulations utilized the COMPASSIII force field.^41^ Based on the results of the relaxation calculation, we obtained a PBC cell with a side length of 17.0–17.5 Å. The procedure was repeated 20 times, each time using the initial structure for a reactive MD calculation, and the results of the 20 iterations were averaged. The temperature conditions were 1200, 1500, and 2000 K, and the pressure was 101 kPa. In all cases, MD simulations were performed at constant pressure and temperature.

PFP version 6.0.0 (CRYSTAL_U0 calculation mode)^24^ in Matlantis, which is software for service style material discovery, was used for the NNP. PFP, as a pretrained NNP model, enables calculations by specifying the version and mode. An example of PFP application in the polymerization of hydrocarbon-based polymers has also been included.^42^ For NNP-MD, MD was carried out in the Atomic Simulation Environment module.^43^ The time step was set to 1.0 fs/step, based on results indicating that this step size provided results consistent with those obtained at 0.25 fs/step concerning monomer yields (Figure S6). NNP-MD simulations were extended until no further increase was observed in the decomposition rate (Figure S7). ReaxFF-MD used the ReaxFF potential,^44^ and the charges were calculated using the charge equilibration method (*Q*_eq_),^45^ which was applied for pyrolysis and polymer degradation.^46,47^ MD was performed on a large-scale atomic/molecular massively parallel simulator (LAMMPS).^48^ The time step was set to 0.25 fs/step, based on results indicating that a step value below 0.5 fs/step is necessary for reliable simulations with Chenoweth’s ReaxFF potential.^49^ The time scale was 7.5 ns at all temperatures. The accuracy of the calculation method was determined according to whether the amount and ratio of polystyrene decomposition products from the simulations were consistent with the amount and ratio of the decomposition products from the experiments. The target decomposition products were styrene (a monomer), 2,4-diphenyl-1-butene (a dimer), 2,4,6-triphenyl-1-hexene (a trimer), toluene, EB, αMeSt, and LC, whose formation by thermal decomposition has been reported in various studies.^39,50^ LC was defined as a compound having a molecular weight less than or equal to that of a dimer and not corresponding to any of the degradants under consideration. The weight percentages of degradation products of polystyrene shown in Figures 1, 2, 5, and 7 are listed in Tables S1–S4.

The DFT calculation results illustrated in Figure 6 were obtained using Gaussian 16.^51^ First, a TS with a single imaginary frequency was obtained. Then, based on the structure of the TS, the reaction path followed by the intrinsic reaction coordinate (IRC) was generated. The computational level was ωB97-XD/6-31G(d). All of the ReaxFF and NNP values in Figure 6 are the result of single-point calculations on the structure of the IRC obtained by DFT. The energies shown in Figure 6 are listed in Table S5.

## Data Availability

The data underlying this study are available in this article and Supporting Information.

## Supporting Information Available

Structures at 7.5 ns obtained using ReaxFF at 2000 K (Figures S1); structures at 600 ps obtained using NNP at 1500 K (Figure S2); sampling of the degraded system alone obtained by NNP-MD at 1500 K (Figure S3); changes in the number of monomers starting from an initial structure consisting solely of monomers; the number of monomers decreased rapidly around 250 ps only at 2000 K (Figure S4); correlation graphs at 1500, and 1200 K; refer to Figure 8 in the main text for information on the correlation coefficients (Figure S5); differences in monomer yield based on time step at 2000 K; the red circle corresponds to 1.0 fs/step, whereas the blue circle corresponds to 0.25 fs/step. Both results represent averages of 20 trials (Figure S6); decomposition ratios at 1200, and 1500 K. At 1500 K, a decomposition rate of 88.1 w% was obtained at 1500 ps, whereas at 1200 K, a decomposition rate of 89.6 w% was obtained at 600 ps (Figure S7); the weight percentage of degradation products at 2000 K by using (a) NNP-MD and (b) ReaxFF-MD (Table S1); the weight percentage of degradation products at 1500 K by using (a) NNP-MD and (b) ReaxFF-MD (Table S2); the weight percentage of degradation products of polystyrene including a radical in the initial structure at 1500 K by using (a) NNP-MD and (b) ReaxFF-MD (Table S3); the weight percentage of degradation products of polystyrene including a radical in the initial structure at 1200 K by using (a) NNP-MD and (b) ReaxFF-MD (Table S4); energies of IRC, as shown in Figure 6; units of energy are kcal/mol (Table S5) (PDF)
