# Supplementary material for: Comparison of Computational Methods for Simulating Depolymerization Reaction
Source: ACS Omega. 2025 Feb 4;10(6):5973–80. doi: 10.1021/acsomega.4c09953 (PMC11840784; doi:10.1021/acsomega.4c09953)
Supplement: Supplementary file 1 — ao4c09953_si_001.pdf [file ao4c09953_si_001.pdf]

Supporting Information

Comparison of Computational Methods for Simulating Depolymerization Reactions

Shunsuke Mieda*
Email: mieda.sd@om.asahi-kasei.co.jp
Platform Laboratory for Science & Technology, Asahi Kasei Corporation, 2-1 Samejima, Fuji, Shizuoka 416-8501, Japan

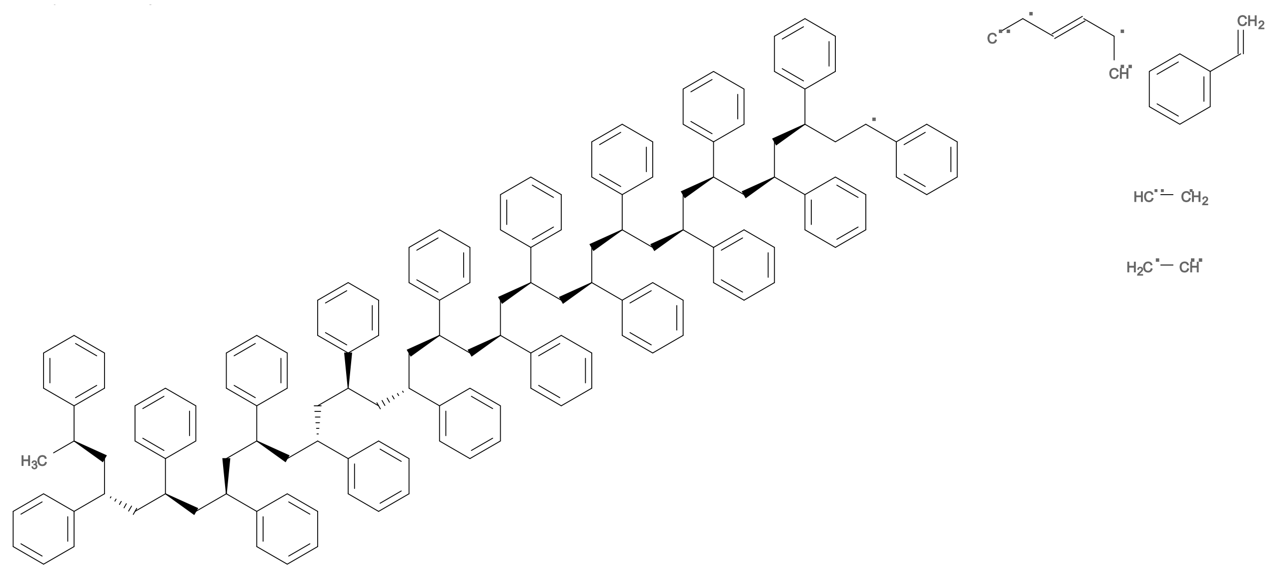

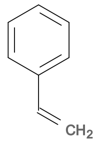

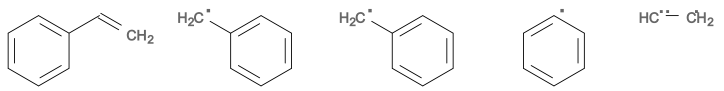

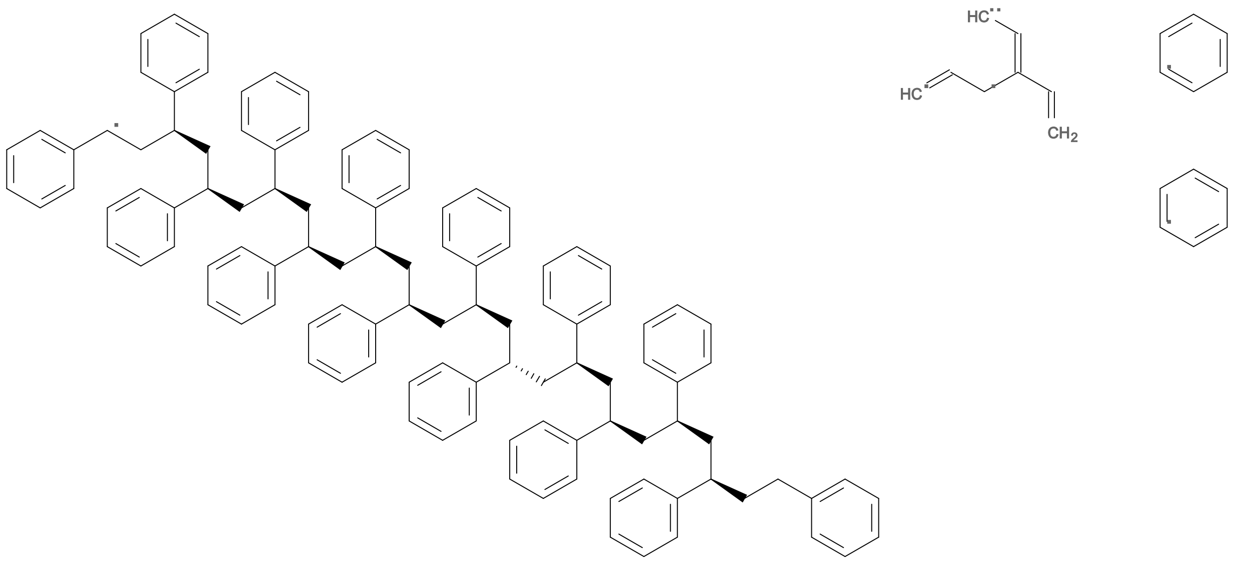

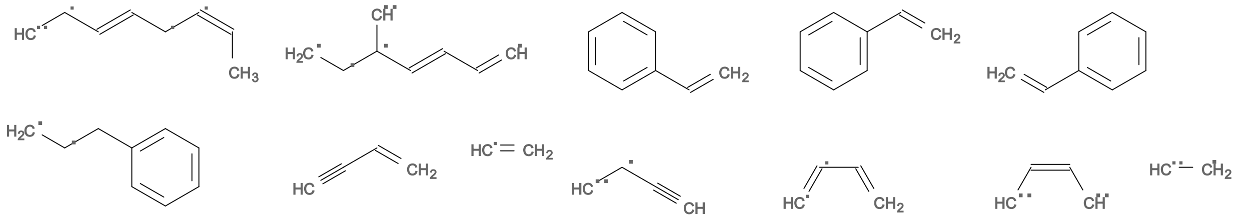

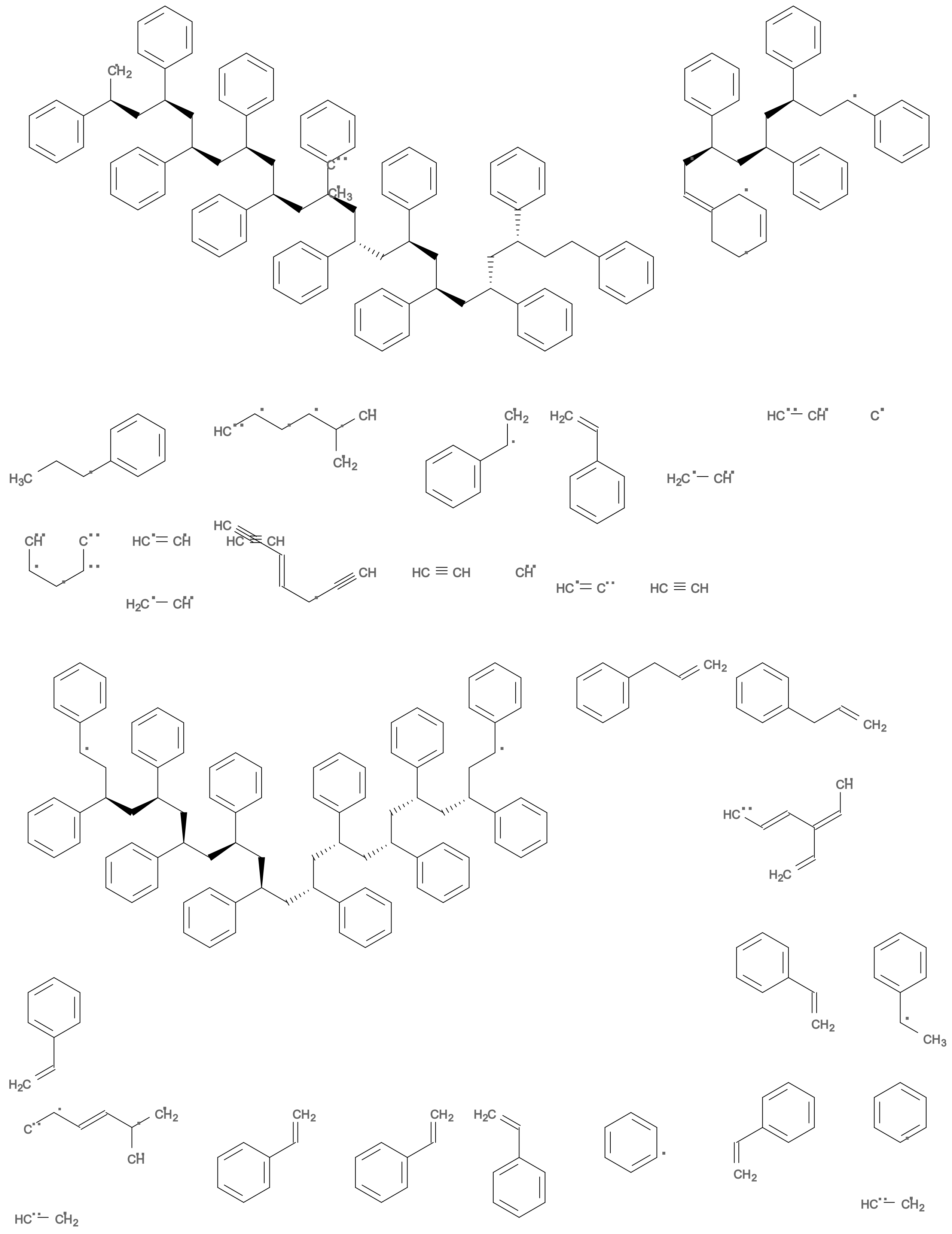

Figure S1. Structures at 7.5 ns obtained using ReaxFF at 2,000 K.

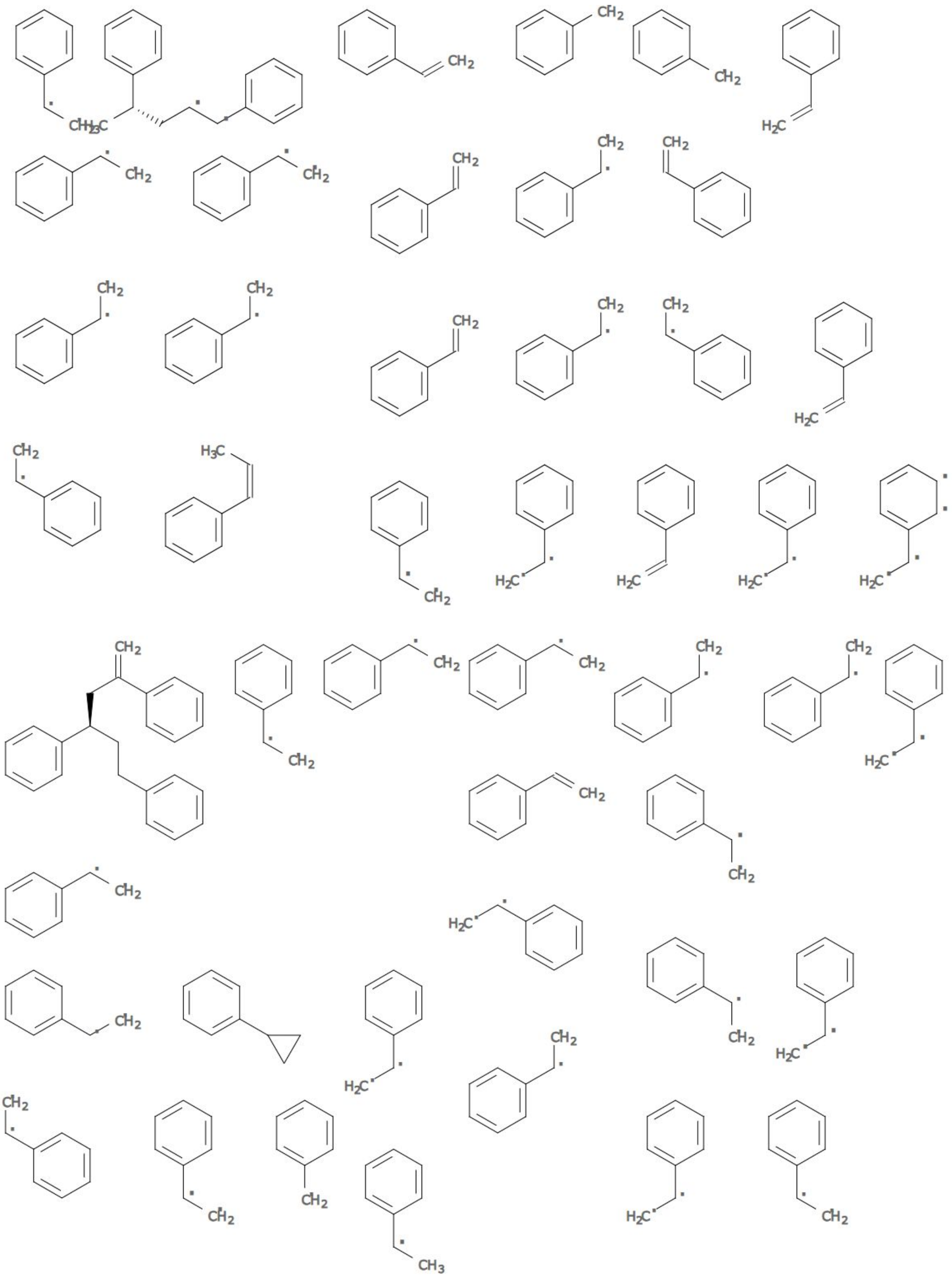

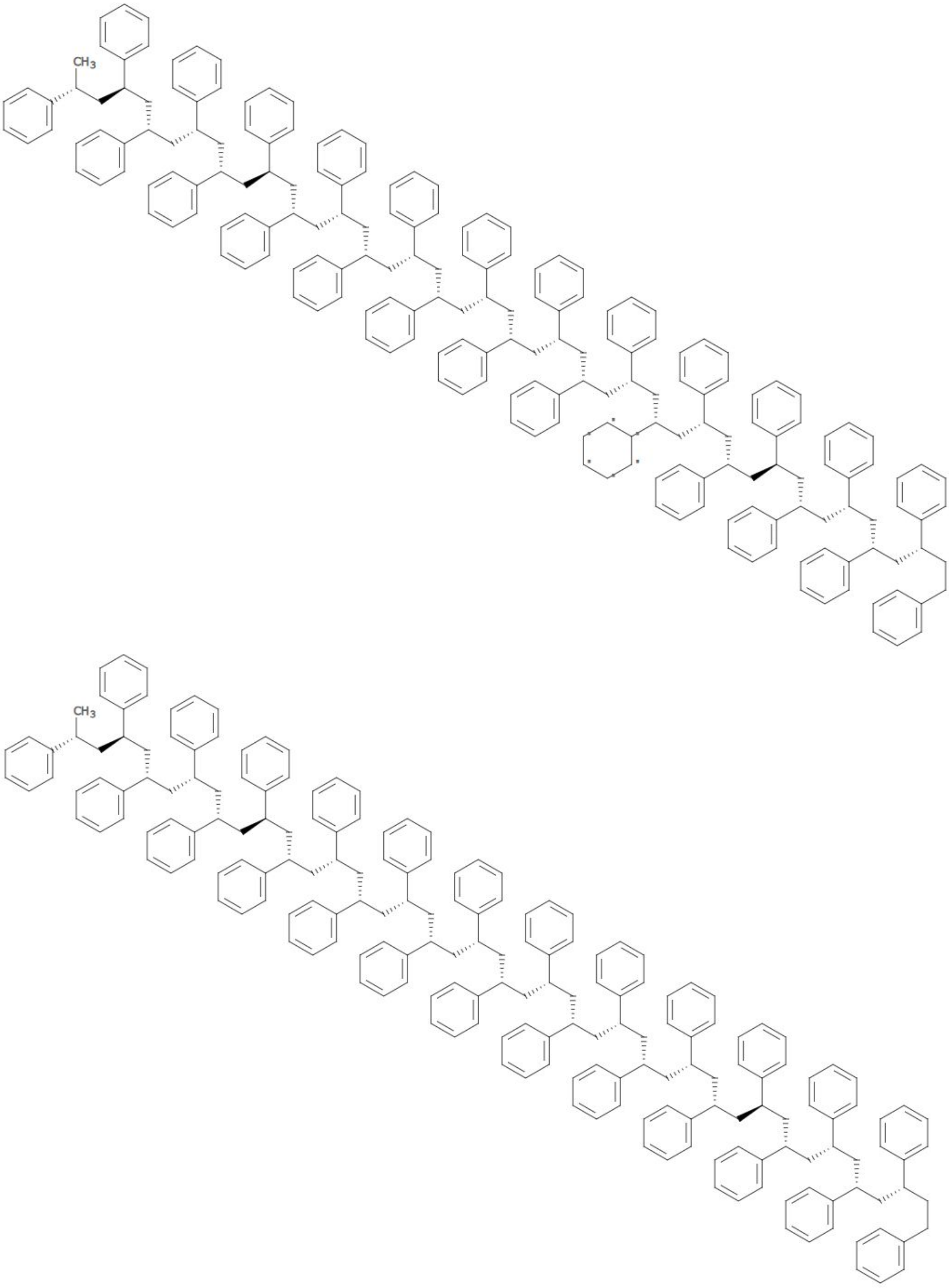

Figure S2. Structures at 600 ps obtained using neural network potential (NNP) at 1,500 K.

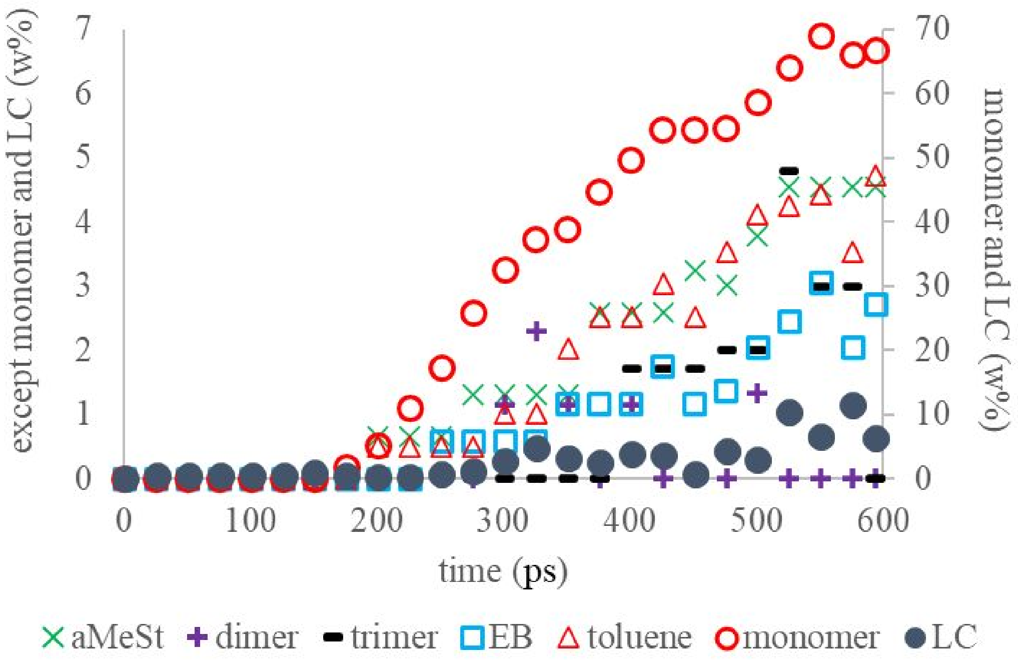

Figure S3. Sampling of the degraded system alone obtained by NNP-MD at 1,500 K.

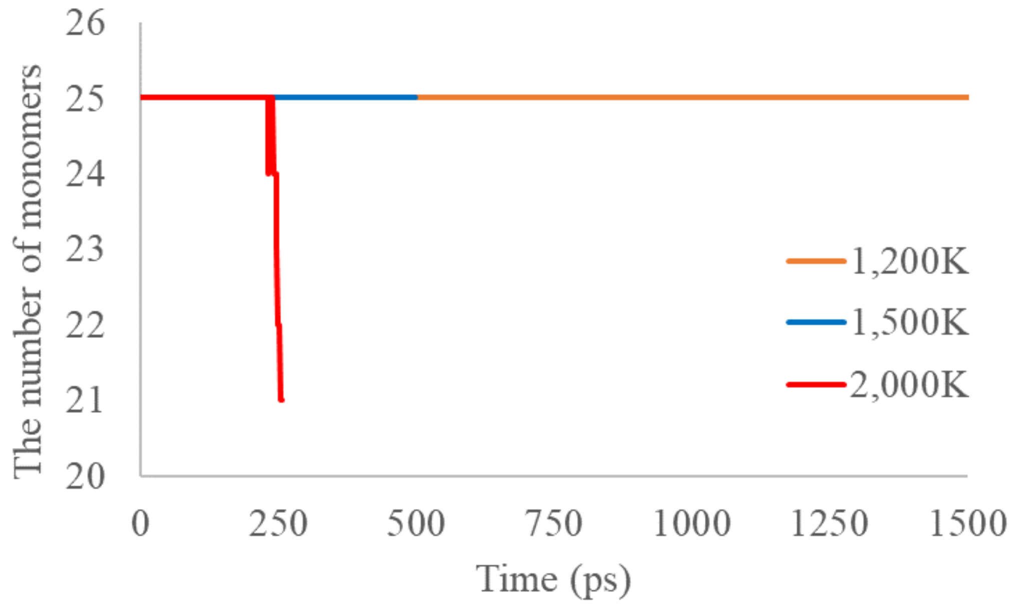

Figure S4. Changes in the number of monomers starting from an initial structure consisting solely of monomers; the number of monomers decreased rapidly around 250 ps only at 2,000 K.

(a)

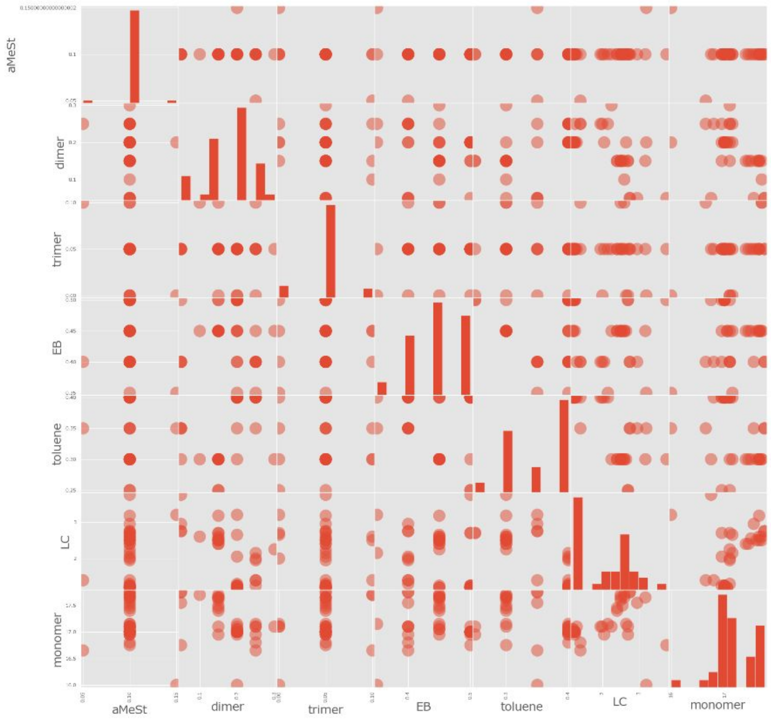

(b)

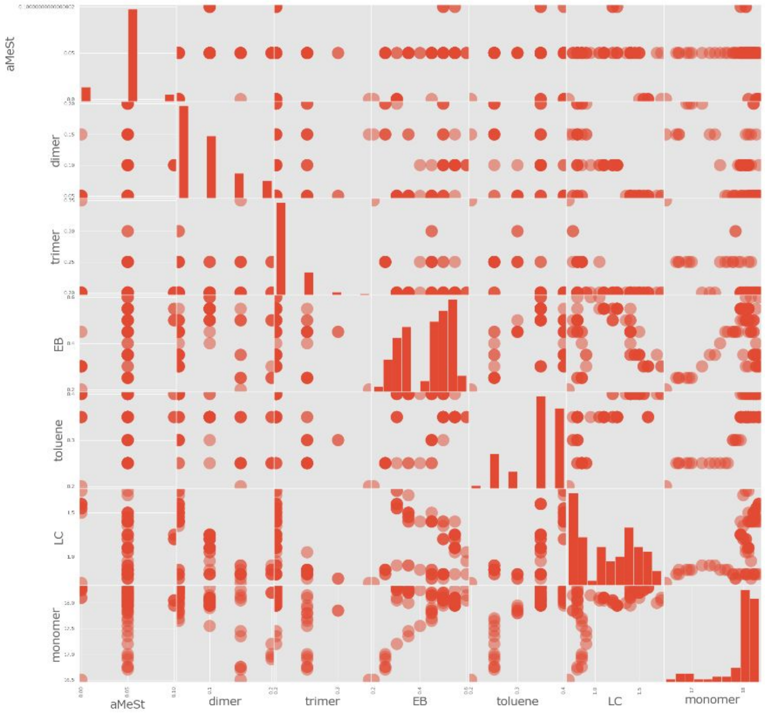

Figure S5. Correlation graphs at 1,500 K and 1,200 K; refer to Figure 8 in the main text for information on the correlation coefficients.

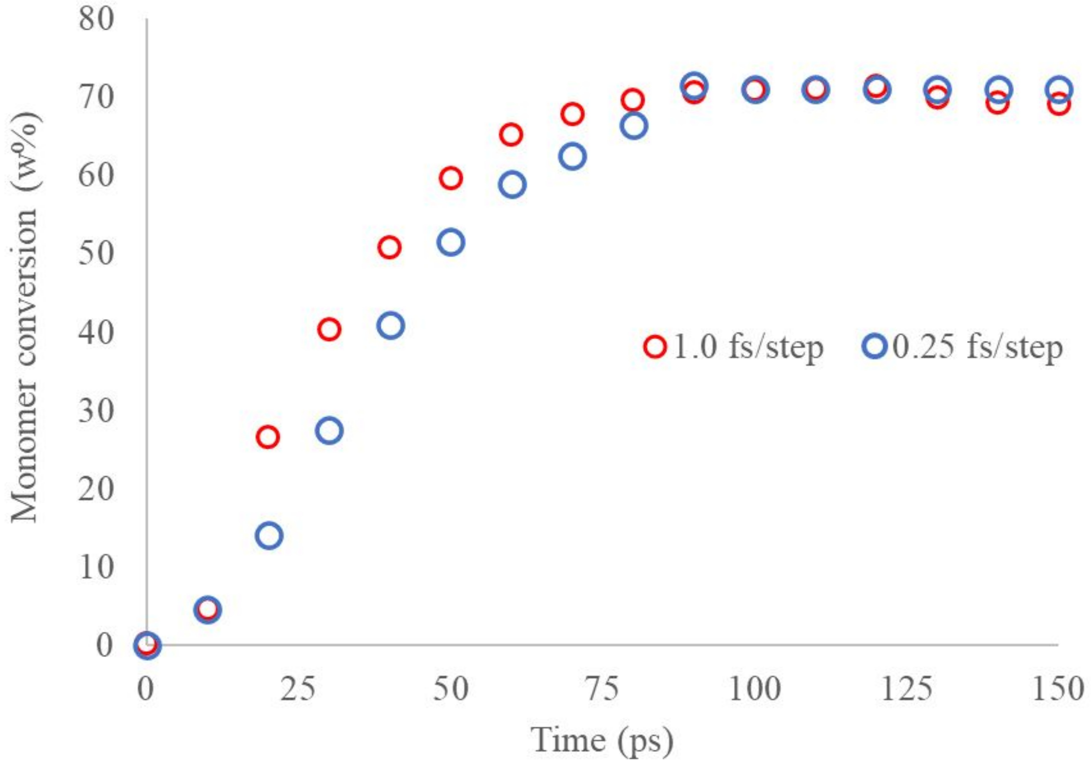

Figure S6. Differences in monomer yield based on timestep at 2,000 K; the red circle corresponds to 1.0 fs/step, whereas the blue circle corresponds to 0.25 fs/step. Both results represent averages of 20 trials.

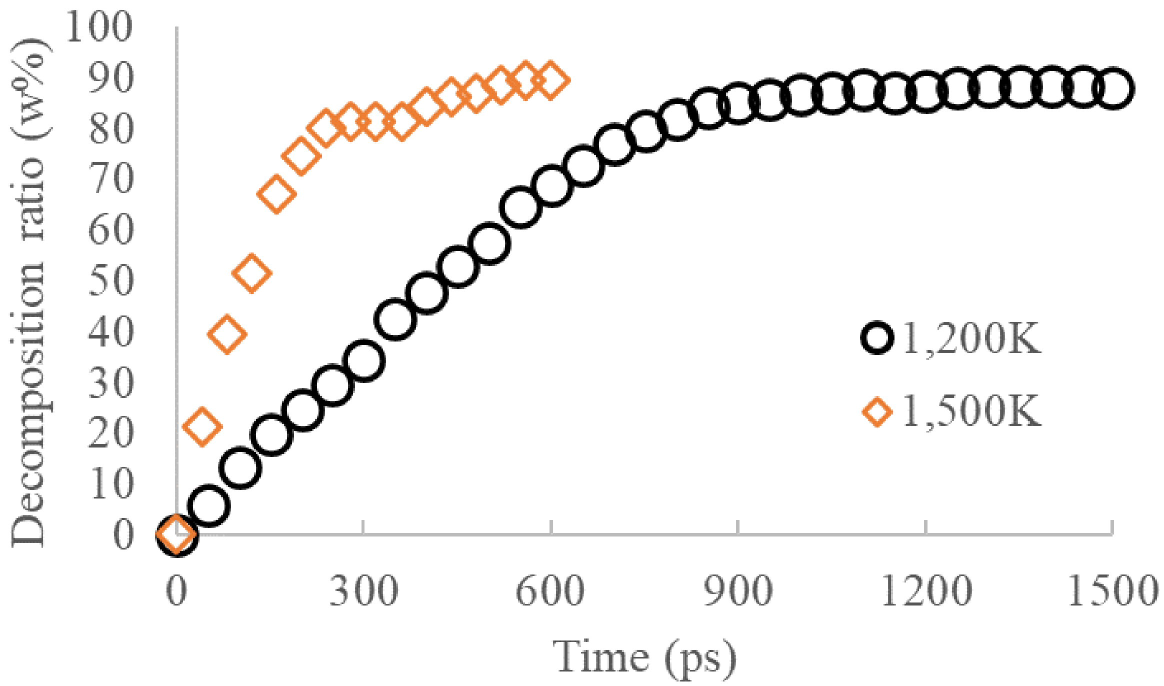

Figure S7. Decomposition ratios at 1,200 K and 1,500 K. At 1,500 K, a decomposition rate of 88.1 w% was obtained at 1,500 ps, whereas at 1,200 K, a decomposition rate of 89.6 w% was obtained at 600 ps.

Table S1(a) The weight percentage of degradation products at 2,000 K by using NNP-MD for Figure 1(a).

time (in ps)	aMeSt	dimer	monomer	trimer	LC	EB	toluene
0	0.00	0.00	0.00	0.00	0.00	0.00	0.00
10	0.64	1.12	4.30	1.68	1.65	0.38	0.00
20	1.27	1.87	24.87	1.12	8.05	0.95	0.33
30	2.12	0.75	37.77	1.12	10.63	1.91	1.16
40	2.55	1.50	47.49	1.12	12.76	2.67	1.65
50	3.18	1.87	55.72	0.56	16.07	2.86	2.48
60	3.18	1.12	60.95	0.56	16.07	3.43	3.31
70	2.97	1.50	63.38	0.56	17.48	3.24	3.14
80	3.18	0.75	65.06	0.56	17.13	3.43	3.64
90	3.18	1.12	66.00	0.56	17.85	3.43	3.64
100	3.18	0.75	66.37	0.56	18.04	3.05	3.80
110	3.18	0.75	66.56	0.56	18.52	3.24	3.80
120	3.18	1.12	66.75	0.56	18.64	3.24	3.80
130	3.18	0.75	65.44	0.56	20.91	3.24	3.64
140	3.18	0.75	64.88	0.56	22.90	2.86	3.47
150	3.18	0.75	64.69	0.56	22.70	3.05	3.47
160	2.97	0.75	64.88	0.56	23.49	2.48	3.47
170	2.76	0.75	59.46	0.56	29.46	2.48	3.14
180	2.76	0.75	52.72	0.56	37.84	1.52	2.65
190	2.33	0.75	46.55	0.56	44.53	1.72	2.81
200	2.12	0.37	40.20	0.56	52.69	1.14	2.32
210	1.49	0.37	28.23	0.56	65.53	1.52	1.82
220	1.49	0.37	27.30	0.56	67.04	0.95	1.82
230	1.27	0.37	22.62	0.56	72.33	1.14	1.65
240	1.49	0.37	20.94	0.56	74.13	1.14	1.32
250	1.49	0.75	20.01	0.00	76.74	0.19	0.83

Table S1(b) The weight percentage of degradation products at 2,000 K by using ReaxFF-MD for Figure 1(b).

time (in ps)	aMeSt	dimer	monomer	trimer	EB	toluene	LC
0	0.00	0.00	0.00	0.00	0.00	0.00	0.00
500	5.90	0.80	31.58	1.20	1.63	1.24	8.23
1000	5.67	0.80	30.58	1.20	1.43	1.41	9.48
1500	5.44	0.80	30.38	1.20	1.22	1.41	10.14
2000	5.44	0.80	30.18	1.20	1.22	1.41	10.34
2500	4.99	0.80	29.78	1.20	1.22	1.59	11.02
3000	4.99	0.80	29.38	1.20	1.43	1.41	11.39
3500	4.76	0.80	29.78	1.20	1.22	1.41	11.63
4000	4.76	0.80	29.18	1.20	1.22	1.59	12.73
4500	4.76	0.80	28.98	1.20	1.43	1.41	13.12
5000	4.76	0.80	28.78	1.20	1.22	1.59	13.34
5500	4.76	0.80	28.78	1.20	1.43	1.41	13.32
6000	4.76	0.80	28.38	1.20	1.22	1.59	13.74
6500	4.76	0.80	28.18	1.20	1.22	1.41	14.12
7000	4.99	0.80	27.38	1.20	1.22	1.59	14.92
7500	4.54	0.80	26.78	1.20	1.22	1.41	15.75

Table S2(a) The weight percentage of degradation products at 1,500 K by using NNP-MD for Figure 2(a).

time (in ps)	aMeSt	dimer	monomer	trimer	LC	EB	toluene
0	0.00	0.00	0.00	0.00	0.00	0.00	0.00
20	0.00	0.00	0.00	0.00	0.54	0.00	0.00
40	0.00	0.00	0.00	0.00	0.54	0.00	0.00
60	0.00	0.00	0.00	0.00	0.54	0.00	0.00
80	0.00	0.00	0.00	0.00	0.54	0.00	0.00
100	0.00	0.00	0.00	0.00	0.54	0.00	0.00
120	0.00	0.00	0.00	0.60	0.54	0.00	0.00
140	0.00	0.40	0.00	0.00	0.57	0.00	0.00
160	0.00	0.00	0.00	0.00	0.94	0.00	0.00
180	0.00	0.00	0.80	0.00	0.61	0.00	0.18
200	0.23	0.00	1.80	0.00	0.70	0.00	0.18
220	0.23	0.00	3.20	0.00	0.80	0.00	0.18
240	0.23	0.00	5.20	0.00	0.97	0.20	0.18
260	0.45	0.00	6.99	0.00	1.12	0.20	0.18
280	0.45	0.00	9.59	0.00	1.72	0.20	0.18
300	0.45	0.40	11.39	0.00	1.49	0.20	0.35
320	0.45	0.80	12.59	0.60	2.01	0.20	0.35
340	0.45	0.40	13.59	0.00	1.74	0.41	0.71
360	0.68	0.00	13.99	0.00	1.77	0.41	0.88
380	0.91	0.00	15.79	0.00	1.92	0.41	0.88
400	0.91	0.40	17.39	0.60	2.07	0.41	0.88
420	0.91	0.00	18.19	0.60	2.51	0.61	0.88
440	1.13	0.00	19.19	0.60	2.83	0.61	1.06
460	0.91	0.00	18.79	0.60	5.76	0.41	1.06
480	0.91	0.00	20.38	1.20	6.04	0.41	1.06
500	1.13	0.00	22.18	1.80	7.39	0.61	1.24
520	1.13	0.00	22.78	1.80	9.12	0.61	1.06
540	1.13	0.00	21.78	1.80	12.31	1.02	0.88
560	1.13	0.40	23.98	1.80	13.23	0.61	0.88
580	0.91	0.40	21.58	1.80	16.69	0.81	0.71
600	0.91	0.00	22.58	1.80	17.30	0.41	0.71

Table S2(b) The weight percentage of degradation products at 1,500 K by using ReaxFF-MD for Figure 2(b).

time (in ps)	aMeSt	dimer	monomer	trimer	EB	toluene	LC
0	0.00	0.00	0.00	0.00	0.00	0.00	0.00
500	0.68	0.00	1.80	0.00	1.02	0.18	1.16
1000	0.68	0.40	2.60	0.60	0.81	0.18	1.67
1500	0.91	0.40	2.20	0.60	0.61	0.18	2.29
2000	1.13	0.40	2.60	0.60	0.61	0.18	1.66
2500	1.13	0.40	2.60	0.60	0.61	0.18	1.66
3000	1.13	0.80	2.80	0.60	0.61	0.18	1.70
3500	1.13	0.40	2.80	0.60	0.61	0.18	1.70
4000	1.13	0.40	2.60	0.60	0.61	0.18	1.90
4500	1.13	0.40	2.60	0.60	0.81	0.18	1.91
5000	1.13	0.40	2.60	0.60	0.61	0.18	2.28
5500	1.13	0.40	2.60	0.60	0.41	0.18	2.75
6000	1.13	0.00	2.40	0.60	0.41	0.18	2.52
6500	1.13	0.40	2.60	0.60	0.41	0.18	2.32
7000	1.36	0.00	2.40	0.60	0.41	0.18	2.29
7500	1.13	0.80	2.40	0.60	0.41	0.18	2.68

Table S3(a) The weight percentage of degradation products of polystyrene including a radical in the initial structure at 1,500 K by using NNP-MD for Figure 5(a).

time (in ps)	aMeSt	dimer	monomer	trimer	LC	EB	toluene
0	0.00	0.00	0.00	0.00	0.00	0.00	0.00
20	0.00	0.40	9.79	0.00	1.32	0.00	0.18
40	0.00	0.80	17.99	0.00	2.37	0.00	0.18
60	0.00	0.80	26.58	0.00	3.05	0.20	0.53
80	0.00	0.80	33.97	0.00	4.01	0.20	0.53
100	0.00	1.20	38.97	0.00	4.59	0.41	0.53
120	0.23	1.20	44.17	0.00	4.99	0.20	0.71
140	0.23	1.60	50.76	0.00	5.52	0.20	0.71
160	0.23	2.00	56.56	0.60	6.40	0.41	0.71
180	0.23	2.80	59.35	0.60	7.32	0.41	0.71
200	0.23	1.60	62.35	1.20	7.49	1.02	0.71
220	0.23	1.60	65.55	1.80	6.98	1.43	1.24
240	0.23	2.00	66.55	1.20	7.09	1.63	1.24
260	0.45	2.00	67.75	0.60	7.22	1.83	1.41
280	0.45	1.60	67.95	0.60	7.22	2.04	1.41
300	0.45	1.60	67.95	0.60	7.25	2.04	1.41
320	0.45	1.60	67.95	0.60	7.22	2.04	1.41
340	0.45	1.60	67.95	0.60	7.22	2.04	1.41
360	0.45	1.60	68.15	0.60	7.22	1.83	1.41
380	0.45	1.60	68.35	0.60	7.23	1.63	1.41
400	0.45	2.00	68.35	0.60	9.81	1.63	1.41
420	0.45	2.00	68.35	0.00	10.45	1.63	1.41
440	0.45	2.40	68.35	0.60	11.49	1.83	1.06
460	0.45	1.20	67.75	0.60	14.31	2.04	0.88
480	0.45	1.20	69.55	0.60	12.08	1.83	1.06
500	0.45	1.20	69.95	0.60	12.11	1.83	1.06
520	0.45	1.20	70.55	0.60	12.83	1.83	1.06
540	0.45	1.20	70.75	1.20	13.49	1.83	1.06
560	0.45	0.40	70.94	0.60	14.30	1.63	1.24
580	0.45	0.40	70.94	0.60	14.30	1.63	1.24
600	0.45	1.20	70.55	0.60	13.88	1.83	1.06

Table S3(b) The weight percentage of degradation products of polystyrene including a radical in the initial structure at 1,500 K by using ReaxFF-MD for Figure 5(b).

time (in ps)	aMeSt	dimer	monomer	trimer	EB	toluene	LC
0	0.00	0.00	0.00	0.00	0.00	0.00	0.00
500	0.23	0.00	2.40	0.00	0.41	0.00	1.43
1000	0.23	0.40	4.40	0.00	0.41	0.00	1.66
1500	0.23	0.40	4.80	0.00	0.41	0.00	1.90
2000	0.23	0.40	5.40	0.00	0.41	0.00	1.95
2500	0.23	0.40	5.20	0.00	0.41	0.00	2.15
3000	0.23	0.40	5.40	0.00	0.41	0.18	2.17
3500	0.45	0.40	5.40	0.00	0.41	0.18	2.19
4000	0.45	0.40	5.40	0.00	0.41	0.18	2.19
4500	0.45	0.40	5.40	0.00	0.41	0.18	2.19
5000	0.45	0.40	5.80	0.00	0.41	0.18	2.25
5500	0.45	0.40	5.80	0.00	0.41	0.18	2.25
6000	0.45	0.40	5.80	0.00	0.41	0.18	2.25
6500	0.45	0.40	5.80	0.00	0.41	0.18	2.25
7000	0.45	0.40	5.80	0.00	0.41	0.18	2.25
7500	0.45	0.40	5.80	0.00	0.41	0.18	2.25

Table S4(a) The weight percentage of degradation products of polystyrene including a radical in the initial structure at 1,200 K by using NNP-MD for Figure 7(a).

time (in ps)	aMeSt	dimer	monomer	trimer	LC	EB	toluene	tot
0	0.00	0.00	0.00	0.00	0.00	0.00	0.00	0.00
50	0.00	0.00	4.80	0.00	0.90	0.00	0.00	5.69
100	0.00	0.00	10.79	0.60	1.75	0.00	0.00	13.14
150	0.00	0.00	16.59	0.60	2.59	0.00	0.00	19.78
200	0.00	0.00	21.38	0.60	2.56	0.00	0.18	24.72
250	0.00	0.00	25.58	0.60	2.89	0.00	0.35	29.42
300	0.00	0.00	29.78	1.20	3.21	0.00	0.35	34.54
350	0.00	0.00	37.17	1.20	3.76	0.00	0.35	42.49
400	0.00	0.00	41.97	1.20	4.12	0.00	0.35	47.65
450	0.00	0.00	46.76	1.20	4.49	0.00	0.35	52.80
500	0.00	0.00	50.56	1.80	4.77	0.00	0.35	57.49
550	0.00	0.80	54.96	3.00	5.57	0.00	0.35	64.67
600	0.00	1.60	58.55	2.40	5.90	0.00	0.35	68.80
650	0.00	0.80	62.15	2.40	6.17	0.81	0.53	72.86
700	0.00	1.20	65.15	3.00	6.03	0.81	0.71	76.90
750	0.23	1.20	66.95	3.00	6.20	1.02	0.88	79.48
800	0.23	1.60	68.75	2.40	6.78	1.22	0.88	81.86
850	0.23	0.40	70.75	3.00	6.88	1.83	0.88	83.96
900	0.23	0.80	71.54	3.00	6.57	1.83	1.06	85.04
950	0.23	1.20	72.34	2.40	6.28	2.04	1.24	85.73
1000	0.23	1.20	72.94	2.40	6.34	2.24	1.24	86.59
1050	0.23	0.80	72.34	2.40	7.74	2.24	1.24	86.99
1100	0.23	0.80	72.34	3.00	7.74	2.24	1.24	87.59
1150	0.23	0.80	71.74	2.40	8.34	2.24	1.24	86.99
1200	0.45	0.80	72.14	2.40	8.16	2.04	1.24	87.23
1250	0.23	0.40	72.34	2.40	9.27	2.04	1.41	88.09
1300	0.23	0.40	72.74	2.40	9.29	1.83	1.41	88.31
1350	0.23	0.40	73.14	2.40	9.30	1.43	1.41	88.31
1400	0.23	0.40	71.94	2.40	10.50	1.43	1.41	88.31
1450	0.00	0.40	72.94	2.40	9.93	1.22	1.41	88.31
1500	0.23	0.40	72.74	2.40	9.70	1.22	1.41	88.10

Table S4(b) The weight percentage of degradation products of polystyrene including a radical in the initial structure at 1,200 K by using ReaxFF-MD for Figure 7(b).

time (in ps)	aMeSt	dimer	monomer	trimer	EB	toluene	LC
0	0.00	0.00	0.00	0.00	0.00	0.00	0.00
500	0.23	0.40	2.80	0.00	0.20	0.18	1.19
1000	0.23	0.00	2.60	0.00	0.41	0.18	1.61
1500	0.23	0.00	2.40	0.00	0.61	0.18	1.61
2000	0.23	0.00	2.60	0.00	0.41	0.18	1.75
2500	0.23	0.00	2.60	0.00	0.41	0.18	1.75
3000	0.23	0.00	2.60	0.00	0.41	0.18	1.75
3500	0.23	0.00	2.60	0.00	0.41	0.18	1.75
4000	0.23	0.00	2.60	0.00	0.20	0.18	1.95
4500	0.00	0.00	2.60	0.00	0.20	0.18	2.18
5000	0.23	0.00	2.80	0.00	0.20	0.00	1.93
5500	0.23	0.00	2.60	0.00	0.41	0.00	1.93
6000	0.23	0.00	2.60	0.00	0.41	0.00	1.93
6500	0.23	0.00	2.60	0.00	0.20	0.00	2.13
7000	0.23	0.00	3.00	0.00	0.41	0.00	1.96
7500	0.23	0.00	3.20	0.00	0.20	0.00	2.17

Table S5. Energies of IRC as shown in Figure 6; units of energy are kcal/mol.

	DFT	ReaxFF	NNP
1	0.000	4.290	0.000
2	0.028	4.317	0.052
3	0.061	4.350	0.106
4	0.099	4.389	0.164
5	0.141	4.430	0.225
6	0.191	4.474	0.289
7	0.247	4.520	0.358
8	0.311	4.567	0.430
9	0.383	4.619	0.507
10	0.464	4.668	0.586
11	0.555	4.701	0.660
12	0.661	4.697	0.723
13	0.794	4.601	0.775
14	0.975	4.331	0.829
15	1.237	3.776	0.927
16	1.626	2.783	1.118
17	2.193	1.042	1.450
18	3.097	0.000	2.029
19	4.664	0.903	3.140
20	7.080	3.255	4.923
21	10.213	7.211	7.225
22	13.833	12.678	9.798
23	17.693	19.213	12.387
24	21.509	25.819	14.666
25	24.939	31.282	16.275
26	27.577	34.466	16.894
27	29.113	34.792	17.057
28	29.245	28.503	18.334
29	28.522	24.036	19.107
30	27.631	20.491	19.575
31	26.738	18.082	19.754
32	25.912	16.574	19.703
33	25.173	15.672	19.480
34	24.524	15.349	19.146
35	23.964	15.003	18.751

36	23.480	14.747	18.337
37	23.062	14.551	17.924
38	22.701	14.401	17.523
39	22.395	14.279	17.140
40	22.136	14.182	16.793
41	21.918	14.107	16.477
42	21.734	14.052	16.193
43	21.580	14.019	15.943
44	21.453	13.995	15.730
45	21.346	13.987	15.553
46	21.256	13.984	15.412
47	21.179	13.980	15.307
48	21.113	13.972	15.237
49	21.054	13.958	15.193
50	21.000	13.938	15.167
51	20.948	13.911	15.153
52	20.899	13.877	15.147
53	20.852	13.840	15.144
54	20.807	13.799	15.141
55	20.762	13.757	15.139
56	20.718	13.714	15.139
57	20.676	13.669	15.138
58	20.633	13.623	15.136
59	20.590	13.576	15.131
60	20.548	13.528	15.124
61	20.506	13.481	15.118
62	20.465	13.434	15.112
63	20.423	13.387	15.106
64	20.381	13.340	15.098
65	20.339	13.295	15.089
66	20.296	13.249	15.078
67	20.254	13.206	15.068
68	20.211	13.166	15.059
69	20.168	13.128	15.052
70	20.124	13.093	15.047
71	20.080	13.059	15.044
72	20.036	13.025	15.040

73	19.992	12.991	15.035
74	19.947	12.957	15.031
75	19.902	12.921	15.027
76	19.858	12.885	15.026
77	19.812	12.850	15.027
